# Peculiarities of the T Cell Immune Response in COVID-19

**DOI:** 10.3390/vaccines10020242

**Published:** 2022-02-04

**Authors:** Dmitry Kudlay, Ilya Kofiadi, Musa Khaitov

**Affiliations:** 1 NRC Institute of Immunology FMBA of Russia, 115522 Moscow, Russia; da.kudlay@nrcii.ru (D.K.); mr.khaitov@nrcii.ru (M.K.); 2 Department of Pharmacology, I.M. Sechenov First Moscow State Medical University (Sechenov University), 119991 Moscow, Russia; 3 Department of Immunology, Pirogov Russian National Research Medical University, 117997 Moscow, Russia

**Keywords:** T cell, immune response, COVID-19, SARS-CoV-2

## Abstract

Understanding the T cell response to SARS-CoV-2 is critical to vaccine development, epidemiological surveillance, and control strategies for this disease. This review provides data from studies of the immune response in coronavirus infections. It describes general mechanisms of immunity, its T cell components, and presents a detailed scheme of the T cell response in SARS-CoV-2 infection, including from the standpoint of determining the most promising targets for assessing its level. In addition, we reviewed studies investigating post-vaccination immunity in the development of vaccines against COVID-19. This review also includes the peculiarities of immunity in different age and gender groups, and in the presence of a number of factors, for example, comorbidity or disease severity. This study summarizes the most informative methods for assessing the immune response to SARS-CoV-2 infection.

## 1. Introduction

Nowadays, the world is facing a crisis that began in late December 2019 with several cases of pneumonia in Wuhan, China. Patients presented with a fever, dry cough, sore throat, shortness of breath, and fatigue. It was found that samples of body fluids and swabs from the mouth and anal area contained coronavirus. Subsequent sequencing and phylogenetic analysis revealed a new causative agent of respiratory infections: 2019-nCoV, which the International Committee on the Taxonomy of Viruses subsequently named SARS-CoV-2 [1,2].

As more cases were occurring worldwide, in February 2020, the World Health Organization (WHO) called this pathology Coronavirus Disease 2019 (COVID-19) and declared it a pandemic in March 2020. The global outbreak of SARS-CoV-2 has had a major impact on the global economy. The main way people become infected is via airborne transmission. Symptoms may appear 2–14 days after exposure to the virus as indicated for the original Wuhan strain [3].

Details of the human immune response to SARS-CoV-2 are continually being updated. There are separate studies aimed at studying the peculiarities of the immune response in patients with COVID-19, and following immunization against SARS-CoV-2 [4,5,6,7,8,9]. In this regard, we can talk about some of the aspects of the immune response in this pathology with a high degree of confidence. In this review, we summarize the results of studies aiming to investigate the role of the T cell link in the immune response mechanism of SARS-CoV-2.

## 2. General Mechanisms of the Immune Response

The immune response consists of innate immunity capable of recognizing and neutralizing antigens, and acquired immunity activated by direct contact with them. The cellular component of innate immunity is represented by dendritic cells and tissue macrophages, natural killers, and some lymphocyte subpopulations. Humoral innate immunity is represented by the active compounds produced in natural barriers: lysozyme, interferons, the complement system, and inflammatory mediators [10].

Acquired or adaptive immunity is also represented by cellular and humoral components. The essence of humoral immunity is the synthesis of specific antibodies by B cells [11,12]. T cells play a key role in the implementation of the pathway of cellular adaptive immunity [13].

### 2.1. T Cell Response

Initially, T cells develop and mature in the thymus. Then, they enter the bloodstream and are transferred to the peripheral lymphoid organs. Circulation between blood and lymphoid tissue occurs until a cell encounters its specific antigen through antigen-presenting cells (APCs). Mature recirculating T cells that have not yet been exposed to antigen are called naïve T cells. To participate in the adaptive immune response, the naïve T cell must first encounter the antigen. Afterward, cells activate and begin to actively divide, forming clones. Some of the clone cells turn into effector T cells (T helper cells (CD4+), T killer cells (CD8+). CD8+ T cells activated by an antigen mediate the direct killer function of the adaptive immune response. T helper cells activated by an antigen can stimulate other cells involved in adaptive immunity that have already encountered the same antigen. They also promote the phagocytic properties of macrophages by releasing interleukin-2 (IL-2), tumor necrosis factor-α (TNF-α), and interferon-γ (IFN-γ). Other cells are transformed into memory T cells that remain inactive after initial contact with the antigen until they contact with the same antigen again.

### 2.2. Immune Response to Cognate Coronaviruses

In general, the experience of past epidemics caused by cognate coronaviruses suggest a relatively short-lived antibody response and more persistent T cell immunity. It has previously been shown that neutralizing antibodies correlate with the level of protection in MERS-CoV but do not remain in circulation for long. Moreover, both CD4+ and CD8+ T cells were induced after infection, and were detectable even in the absence of virus-specific antibodies [14]. In vitro studies in cell cultures isolated from patients with SARS have shown that T cells specific for SARS-CoV are essential for the recognition and lysis of infected cells, especially in the lungs [15]. A more robust cytotoxic T cell response has been shown to promote protection against SARS-CoV in mice [16,17,18]. In recovered patients, T cell epitopes were identified, and overlapping peptide pools that interact with the full-length SARS-CoV antigens. Among them, those that determined T cell responses against SARS-CoV were distinguished [19,20,21,22,23]. While serum antibody levels decreased in all patients, the cytotoxic T cell activity against S-proteins and N-proteins of SARS-CoV was detected among peripheral blood mononuclear cells (PBMCs) in recovered SARS patients at 1, 2, 4, 6, and even >10 years post-infection [24,25,26,27].

There is also evidence of adaptive immunity to MERS-CoV obtained from studies on samples collected from patients in South Korea [28,29], China [24,30], the United States [31], and Saudi Arabia [32]. Seroconversion in most patients occurred within 2 and 3 weeks after the onset of symptoms [30,32,33].

In patients infected with MERS-CoV, low antibody titer or delayed development was associated with more severe disease or death [28,32,33]. Corman et al. (2016) reported that the levels of IgG and neutralizing antibodies did not correlate with the viral load in the lower respiratory tract, and thus concluded that the presence of antibodies does not lead to virus elimination [32].

In a study by Da Guan et al. (2015) in patients infected with MERS-CoV, analysis of peripheral blood mononuclear cells (PBMCs) isolated 24 days after the onset of disease showed a strong specific T cell response against MERS-CoV S protein [24]. In patients with a fatal outcome, a significant decrease in the level of T lymphocytes was detected [28]. In addition, it was found that IL-12 and IFN-γ levels were significantly lower in patients with fatal outcomes compared to patients who later recovered (*p* < 0.05) [34].

According to published data from Al-Abdallat et al. (2014), in patients (*n* = 7) infected with MERS-CoV, the level of specific antibodies was maintained 13 months after the outbreak of the infection in a Jordanian hospital [35]. However, in a longitudinal study by Alshukairi et al. (2016), only 2 of the 9 patients who recovered after 18 months had specific antibodies to MERS-CoV [36]. In a study by Arabi et al. (2016), only 4 (36.7%) of 11 healthcare professionals who had a history of laboratory-confirmed MERS-CoV infection had anti-viral antibody levels that were detected by enzyme-linked immunosorbent assay (ELISA) on average 381 days after infection [37].

In several studies, it was found that the major histocompatibility complex (MHC) class II presents SARS-CoV peptides to CD4+ T cells [38]. Due to the genetic polymorphism of human leukocyte antigens (HLAs), some haplotypes, such as HLA-B*07, HLA-B*46, HLA-DRB1*12, and HLA-Cw *08, were found to be more susceptible to coronavirus infection while the haplotypes HLA-DRB1*03, HLA-A*02, and HLA-Cw *15 were protected against SARS-CoV infection [38]. In a study by Giamarellos-Bourboulis et al. (2020), it was found that the blood plasma of COVID-19 patients could block HLA-DR expression on CD14 + monocytes, which recovered upon IL-6 inhibition [39]. This reduction in MHC expression is evident in cancer cells, the mechanism by which they evade the immune response by epigenetic modification of calnexin. From this perspective, compounds capable of activating chromatin regulators and increasing the expression of MHC-I may potentially be utilized for COVID-19. Most of the T cell epitopes represented by the MHC complex originate from structural proteins, such as coronavirus S protein and N protein. It has been proven that T cells can be stimulated by 14 epitopes, most of which are located on the SARS-CoV S-protein ORF3 domain [40].

Some SARS-CoV-2 epitopes, including those located within the spike protein, have shown high homology across other human coronaviruses. Memory T and B lymphocytes against seasonal coronaviruses can be re-activated by SARS-CoV-2 infection, producing cross-reactive antibodies and activating T cell-mediated responses. This may have a functional outcome expressed as increased protection for the host; however, it is still debatable [41,42,43]. 

The clinical effect of immunity against seasonal coronaviruses on COVID-19 was shown in the work of Yamaguchi et al. This group studied the titers of antibodies against S-proteins of SARS-CoV-2 and HCoV-OC43 in patients hospitalized with COVID-19, and analyzed the correlation between antibody titers and the severity of each case. This study showed that the high immunity against HCoV-OC43 protects patients hospitalized with COVID-19 from severe and fatal exacerbations [44].

Thus, in the studies of the immune response to cognate coronaviruses, the presence of pronounced T cell and humoral components was observed, with a varying duration between the different studies. However, the antiviral response is characterized by a transient nature, and peripheral memory B cells may not be sufficient to protect against reinfection. Consequently, SARS-CoV-specific CD4+ and CD8+ T cells may play an important role in providing protective immunity against coronaviruses. The pre-existing immunity may soften the clinical outcome of COVID-19 and to some degree may explain the inter-populational differences in infection rates. The results of the presented studies shed light on the pathogenesis of the new coronavirus infection. However, the possibilities of extrapolation and the immunological and epidemiological aspects of the newly discovered facts deserve to be discussed in a special review.

## 3. Immune Response in COVID-19

### 3.1. Overall Immunity in COVID-19

**Figure 1:**
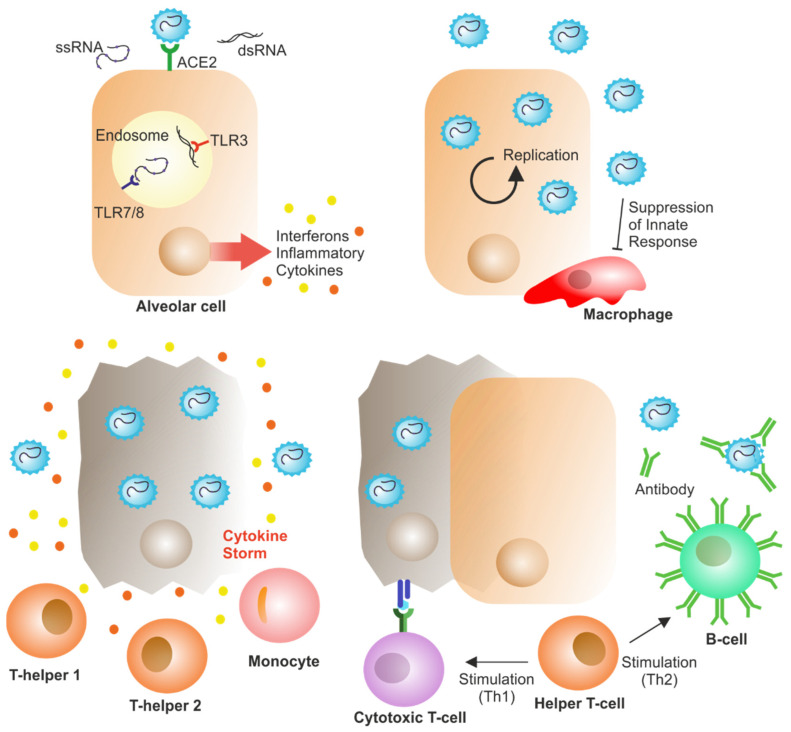
Simplified scheme of the immune response in COVID-19.

When the SARS-CoV-2 virus enters the target cell, the host’s immune system recognizes it as a whole or its surface epitopes, which leads to the activation of the innate and acquired immune response. Infectious agent recognition receptors present on immune cells, mainly toll-like receptors 3, 7, and 8, are the first to identify the virus, resulting in enhanced IFN-γ production, as shown in Figure 1 [45]. There is evidence that SARS-CoV-2 infection leads to an overall decrease in the transcription of antiviral genes due to lower production of type I and III interferons. It has been established that a decrease in the innate antiviral response along with hyperinflammation may be one of the causes of the COVID-19 severity [46]. In addition to decreasing the number of T cells, SARS-CoV-2 infection also increases the depletion of effector T cells, thus decreasing the immune response against the virus [47]. Depletion and loss of function of effector T cells result from increased expression of inhibitory receptors [48].

Note: Infection with SARS-CoV-2 virus by airborne droplets is followed by infection of cells expressing the angiotensin-converting enzyme-2 receptor in the lungs, such as type 2 alveolar cells. The virus suppresses the production of interferons, thus evading innate immunity and then unlimited replication of the virus occurs. Infiltration of monocytes, macrophages, neutrophils, and some other cells leads to an increase in the production of proinflammatory cytokines. In a subset of T-helper cells, stimulation of TH1/TH17 by viral epitopes can lead to increased inflammatory responses. This inflammatory response leads to a “cytokine storm” and consequently to immunopathologies, such as pulmonary edema and pneumonia. Cytotoxic T cells attracted to the site of infection attempt to lyse virus-infected cells in the lungs. B cells also recognize viral proteins and are activated to produce antibodies specific to SARS-CoV-2.

### 3.2. Humoral Immune Response in COVID-19

The humoral response to SARS-CoV-2 is similar to that of other coronavirus infections and includes the production of IgG and IgM and serum IgA [4]. In several studies, specific IgA, IgG, and IgM antibodies against SARS-CoV-2 were detected in infected patients at various stages of the disease. IgG levels were constant over time, with IgM levels beginning to decline on average 3 months after disease onset [40,49]. Gudbjartsson et al. (2020) reported that in individuals with a positive PCR result (*n* = 1797), the G class antibody titer increased within 2 months of diagnosis and remained on the plateau until the end of the study (4 months) [50]. Wajnberg et al. (2020) reported that the vast majority of infected people (*n* = 30082) with mild to moderate COVID-19 have a robust IgG response to spike protein with a stable titer for 5 months [51]. Dan et al. observed the duration of S-IgG detection up to 235 days from the onset of disease, whereas RBD-IgG was detected up to 87 days [8]. The detection of SARS-CoV-2-specific antibodies and T cells at late stages after seroconversion was also confirmed in a large prospective study of the Moscow population [52]. The authors came to the conclusion that albeit the long persistence (up to 300 days), the protective effect of antibody and cellular responses inversely correlated with their magnitude [52]. A study by Tan et al. (2020) found very high levels of IgG and IgM in patients with severe infection. They also reported that subjects with low IgG titer had higher viral clearance than subjects with high titer. The authors suggested that a sustained humoral response is associated with a high degree of disease severity, whereas a weak response is associated with the elimination of the virus [53]. In the work of Zhang et al. (2020) in the pediatric population, in 5 of 6 patients, a humoral response was observed with the production of neutralizing antibodies of IgG and IgM classes against N-protein and the RBD domain [54]. Nevertheless, the available results might seem controversial due to their strong dependence on the study design, the significance of a specific antibody response has been unanimously affirmed by the clinical and scientific community. The detection of neutralizing antibodies later after seroconversion indicates the generation of longer-lived plasma cells that provide protection against reinfection. This confirms that vaccination is a fundamentally sound and correct approach to fighting a new coronavirus infection. However, the level of neutralizing antibodies inevitably decreases over time and at certain point, the adaptive response is preserved in the form of T and B cell memory. Thus, clinical and epidemiological studies relevant to COVID-19 prevention strategies must also consider cellular responses.

### 3.3. Cellular Immune Response in COVID-19

CD4+ T cells are activated in response to SARS-CoV-2 antigens presented by dendritic cells with the MHC class II [55]. Virus-specific CD4+ T cells may differentiate into several different T cell types in response to SARS-CoV-2 and exhibit several auxiliary and effector functions. These include Tfh cells, which assist B cells in promoting the affinity and production of antibodies; Th1 cells, which can perform direct antiviral functions by secreting cytokines and recruiting innate immunity cells; CD4 T-cells, which help CD8 T-cells proliferate and differentiate; CD4-CTL, which can have direct cytotoxic activity against virus-infected cells; and CD4+ T-cells, which produce IL-22, which plays a role in wound healing [4]. SARS-CoV-2 circulates Tfh memory cells [56,57,58].

In patients with severe disease, lymphopenia was observed, in particular a decrease in peripheral blood T-lymphocytes [59]. SARS-CoV-2-specific CD4+ T cells have the strongest association with a reduced severity of COVID-19 disease compared to antibodies and CD8+ T cells [57]. The rapid development of SARS-CoV-2-specific CD4+ T cells leads to a milder disease course and accelerated virus clearance [60].

The prevalence and magnitude of CD4 + T cell responses to SARS-CoV-2 correlate with the expression level of each SARS-CoV-2 protein. S-, M-, and NC-proteins are the most prominent targets for SARS-CoV-2-specific CD4 + T cells [56].

CD8 + T cells are critical for clearing many viral infections due to their ability to kill infected cells. CD8+ T cells recognize antigens presented by MHC class I, which contributes to the release of cytokines and increases their cytotoxic activity [55]. SARS-CoV-2-specific CD8+ T cells show high levels of molecules associated with potent cytotoxic effector functions, such as IFNγ, granzyme B, perforin, and CD107a. It is known that CD8+ T cells do not directly recognize the virus itself but only recognize virus-infected cells, whereas CD4+ T cells respond to antigen-presenting cells. This means that T cells do not prevent infection, but their role is associated with a decrease in the viral load in the host [61]. The strongest response of CD8+ T cells is induced by S-, N-, M-, and ORF3a proteins of SARS-CoV-2 [56]. In general, circulating CD8+ T cells specific for SARS-CoV-2 were detected in fewer cases than CD4+ T cells [56,57,58].

T cells are widely produced in response to SARS-CoV-2 infection and vaccination. CD4+ T cells can be detected as early as day 2–4 after the onset of COVID-19 symptoms [4]. Marcotte et al. (2021) found that the number of S-specific T cells producing IFN-γ reach peak values between 3 and 6 months and persist for up to 15 months [62].

After viral clearance, most effector T cells undergo apoptosis, and also a pool of memory T cells is created that, when restimulated, trigger B cells and other immune cells by producing cytokines, whereas cytotoxic memory T cells help to kill infected cells during subsequent infection [6].

### 3.4. Cytokine Secretion in COVID-19

The immune response during viral infection leads to increased secretion of various cytokines, which is poorly regulated and in some cases can become a serious problem. It has been proven that in the case of infection with SARS-CoV, MERS-CoV, and SARS-CoV-2, overproduction of TNF-α, IL-6, IL-8, IL-1β, and IFN-α and -γ occurs [63,64].

In patients with COVID-19, cytokine and chemokine secretion was increased in several studies and was associated with the development of acute respiratory distress syndrome (ARDS) and death. Increased expression of IL-6, TNF-α, macrophage inflammatory protein 1-α, monocyte-3 chemotaxis protein, granulocyte-macrophage colony-stimulating factor, IL-2, and chemokine ligand-10 was reported along with increased chemokine levels [6].

## 4. Population Peculiarities of the T Cell Immune Response

### 4.1. Disease Severity and Comorbidity

According to a recent systematic review assessing the immune response in COVID-19, adult patients experienced persistent peripheral T cell lymphopenia, which positively correlated with the severity of the disease. People with severe or critical COVID-19 have developed more persistent virus-specific T cell reactions [65].

Yu et al. (2020) used flow cytometry and serological analysis to assess the function of T cells and antibodies in recovering COVID-19 patients. The authors suggested that the T cell response is reduced in the presence of comorbidities [66]. A similar study was conducted by the research team of Wang et al. (2020). CD8+ and CD4+ T cells were found to play an important role in the recovery of patients with critical COVID-19. T cell immunity was reduced in patients with a more severe course of infection, and therefore the authors concluded that the relevant parameters can be used as a prognostic marker in COVID-19 [67].

Thus, it has been shown that parameters of T cell immunity in COVID-19 are associated with the severity of the disease and can be used as markers to predict the course of the disease.

### 4.2. Gender-Specific Peculiarities

Takahashi et al. (2020) studied gender differences in viral load, antibody titers specific for SARS-CoV-2, plasma cytokines, and blood cell phenotyping in subjects with moderate COVID-19 who did not receive immunomodulatory drugs. In the experiment, men had higher plasma levels of innate immune cytokines, such as IL-8 and IL-18, and women had stronger T cell activation than male patients during SARS-CoV-2 infection. The T cell response was negatively correlated with the age of the patients and was associated with a worse outcome in males. In women, higher levels of innate immunity cytokines were associated with the worst course of the disease [68]. A review by Ciarambino et al. (2021) described gender differences in the immune response to COVID-19. It was found that women have a more pronounced inflammatory, antiviral, and humoral immune response compared to men. In addition, it was demonstrated that in elderly patients, the severity of COVID-19 is due to a decrease in T cell immunity [69].

### 4.3. Age Peculiarities and T Cell Response in Children

Cohen et al. (2021) showed that the strength of the CD4+ memory cell T cell response to structural SARS-CoV-2 proteins increases with age, with CD8+ T cell activity increasing with the course of the infectious disease. Infected children had lower CD4+ and CD8+ T-cell responses to structural proteins of SARS-CoV-2 compared to infected adults. The authors also reported comparable T cell polyfunctionality. Compared to adults, children had a lower level of antibodies to β-coronavirus, which is evidence of a different initial immunity. The overall response of follicular helper T cells was greater while monocytes decreased, indicating rapid adaptive coordination of T and B cell responses and different levels of inflammation in children and adults. Thus, decreased prior immunity to β-coronavirus and decreased T cell activation in children may contribute to milder COVID-19 pathogenesis [70]. Bajaj et al. (2020) obtained similar data [71] In children, an increase in inflammatory markers was detected: IL-6, IL-1, C-reactive protein, and procalcitonin in serum. A case of the development of a cytokine storm in a 14-year-old boy who then received an IL-1 receptor antagonist with a successful outcome was described while the level of the T cell response remained moderate [72].

### 4.4. Other Peculiarities

The effect of the nutrition type on T cell immunity, including in COVID-19, has been described. Thus, Hirschberger et al. (2021) studied the effect of a very low-carbohydrate diet on T cell immunity in an in vitro model on human T cells and in a study in healthy volunteers. According to the results of the study, the authors demonstrated that ketone bodies formed during a very low-carbohydrate diet enhance the response of CD4+ and CD8+ cells [73]. A review by Cadler (2021) provides information suggesting that both obesity and malnutrition hurt the immune response to COVID-19 and may be factors contributing to the severe course of the disease [74]. In another review, Cadler (2020) showed that deficiencies in vitamins A, B_6_, B_12_, folate, C, D, and E may also be contributing factors [75]. However, these questions require further investigation and confirmation in independent studies. For example, Zelzer et al. point out that there is no clear evidence that serum concentrations of vitamin D metabolites and the vitamin D metabolite ratio affect the course and clinical outcome of COVID-19 [76].

The article by Harald Mangge points out the important role of kynurenine, an intermediate product of tryptophan metabolism in its biological transformation into nicotinic acid. The tryptophan pathway with the formation of kynurenine metabolites plays a crucial role in the mechanisms of immune regulation and negative control of immune inflammation. Alterations in this pathway disturb an effective immune response and the kynurenine plasma concentration may be a promising marker of fatal outcomes in COVID-19 infection [77].

## 5. Vaccine-Induced Immunity to SARS-CoV-2

Observational studies of vaccines based on messenger ribonucleic acid (mRNA), adenoviral vector, virus proteins, and inactivated viruses have demonstrated that even with some differences in efficacy, all vaccine platforms have a significant positive impact on reducing the severity of the disease and the risk of infection [78,79,80,81]. Nevertheless, as the effect of vaccines is discussed mostly with regard to the antibody response, a vaccine capable of activating T cell memory would be beneficial due to the induction of long-lasting immunity. It has been shown that patients who recovered from SARS-CoV possess memory T cells that are reactive to the N protein of SARS-CoV 17 years after the outbreak of SARS in 2003 [82].

The protective role of vaccine-induced T cells may lead to higher efficacy of the vaccine against different variants of the virus, including the variants of concern with a worldwide spread. Despite partial evasion of antibodies, different variants of the virus (strains) often cause only mild or asymptomatic disease in fully vaccinated individuals. This can be accounted for by the inability of the virus to completely elude S-protein-specific T cells [83,84]. Analysis of the effect of virus mutations on the function of T cell recognition showed that in most vaccinated individuals, all variants of the virus are recognized by S-protein-specific T cells induced by mRNA or adenoviral vector vaccines [85,86,87,88]. At the same time, it has been shown that long-term COVID19 in immunocompromised hosts may result in the appearance of new variants of SARS-Cov2 virus with changed properties, which contribute to virus’ ability to escape from cellular immunity. Stanevich et al. report a case of intrahost SARS-Cov2 evolution in which the virus acquired 40 changes [89]. Among the accumulated mutations, 12 reduced or prevented binding of known immunogenic SARS-CoV-2 HLA class I antigens, suggesting that virus immunoediting is largely driven by cytotoxic CD8+ T cell clones. T cell escape mutations acquired within an individual host may give rise to new epidemiologically important variants if they spill over to the general population. Such mutations may affect the efficiency of currently used vaccines. However, the observation of such immunocompromised patients and timely detection of changes that are potentially significant for HLA presentation may help to upgrade vaccines-in-use in accordance with the evolving virus.

Several studies have found that mRNA vaccines induce S-protein-specific T cells, which consequently predominantly produce IL-2 and IFN-γ and recognize different regions of S-protein [90,91]. In a study by Tan et al. (2021), S-protein-specific T cell levels were shown to be equivalent at 3 months in the post-vaccination group and in the group of patients that recovered from SARS-CoV-2 [92]. The peak cellular responses achieved at 3 months postvaccination are followed by contraction, which is consistent with a typical shift from the effector to the memory phase. It was shown that mRNA vaccines produce spike-specific CD8+ and CD4+ T cells, with the latter maintained as memory T cells with T follicular helper (Tfh) and type 1 T helper cell polarization whereas CD8+ T cells continued to decline [93,94].

It should be noted that since T cells recognize short sequences of viral antigens (epitopes) resulting from the processing of viral antigens associated with HLA-class I or HLA-class II molecules, each person will have a different spectrum of T cells, which varies depending on its HLA-class I and class II profile. This is also reflected in the cellular response to vaccine administration [95].

It has been reported that in patients with COVID-19, the number of induced S-protein-specific T cells is also characterized by a high degree of inter-individual variability: at least 10–100 times, regardless of the methods used to detect them [90,96]. Such variability was observed, including in patients older than 80 years or among those undergoing treatment with immunosuppressive drugs [97,98]. In several studies, quantitative differences in cellular immunity levels were observed in both recovered and vaccinated individuals, regardless of their age, gender, or the severity of the SARS-CoV-2 infection [92,99].

Deng et al. (2021) found a significant increase in T cells synthesizing IFN-γ against S, N, and E proteins of SARS-CoV-2 after vaccination [100]. Marcotte et al. (2021) showed that the level of T cells in individuals who received 2 doses of mRNA vaccine is comparable to the peak of the T cell response in COVID-19-recovered patients 3–6 months after the onset of symptoms [8]. Zhu et al. (2020) found that a specific T cell response peaked on day 14 after vaccination with CanSinoBio Ad5 [101]. Folegatti et al. (2020) showed a pronounced T cell response 56 days after vaccination with ChAdOx1 [102]. Sahin et al. (2020) showed that 2 doses of BNT162b1 vaccine elicited robust CD4 + and CD8 + T cell responses and strong antibody responses, with RBD-binding IgG concentrations exceeding those observed in the serum of a group of people who recovered from COVID-19. Martynova et al. (2021) detected a pronounced antigen-specific T cell response 210 days after vaccination with Sputnik V. The team of Tukhvatulin et al. (2021), assessing the safety, tolerability, and immunogenicity of the Sputnik Light vaccine, showed that 96.6% of participants had an antigen-specific T cell response on day 10 after vaccination while its level was higher in persons with pre-existing immunity to SARS-CoV-2 [79].

It is very interesting that vaccination with vaccines based on divergent platforms (mostly mRNA and vector based) provide an even more pronounced effect. In the study of Borobia et al., it was shown that heterologous vaccination induces a robust immune response and mild reactogenicity [103] The heterologous vaccination regimen resulted in the production of specific antibodies and T-cells, the levels of which were significantly higher than after the homologous vector vaccination and higher or comparable in magnitude to the homologous mRNA vaccination regimens. Heterologous vaccination also resulted in significantly higher levels of specific CD8 T cells compared to both homologous regimens [104].

Of particular interest are studies comparing differences in T cell responses after infection and vaccination. Several studies characterizing the CD8+ T cell response and the CD4+ memory T cell response in naïve and convalescent patients after vaccination have now been performed. Analysis of T cells in healthy SARS-CoV-2-naïve and convalescent subjects before and after primary and booster immunization with the mRNA vaccine showed that vaccination induced a rapid antigen-specific CD4+ T cell response in naïve subjects after the first dose. At the same time, the CD8+ T cell response develops gradually. Vaccine-induced responses of Th1 and Tfh cells after the first dose correlated with post-boost CD8+ T cells and neutralizing antibodies, respectively [90]. Compared to natural infection, the early CD8+ T cell pool is characterized by a different distribution of memory T cell populations, which may affect the duration of protective immunity. This difference may be due to the different duration and site of antigen contact and different inflammatory responses after vaccination and infection, as indicated by lower CD38 expression on specific CD8+ early memory T cells after vaccination compared to natural infection [105]. A study by Denhey at al. showed that vaccination and infection induced comparable levels of specific SARS-CoV-2 CD4+ T cells after three months in addition to comparable proportions of specific central CD4+ memory T cells. In contrast, the proportion of specific effector CD4+ memory T cells was significantly lower and specific effector CD4+ memory T cells were higher after infection than after vaccination [106].

Thus, we see that T cell responses are an additional informative criterion that can be used for assessing and comparing the immunogenicity of vaccines.

## 6. Classic and New Methods for Evaluation of the T Cell Immune Response

### 6.1. Flow Cytometry

The reference method used for assessing the composition and differentiation of cell populations is flow cytometry. This technique has certain shortcomings related to the cost and complexity of interpretation for routine practice [107].

MHC multimer staining followed by flow cytometry analysis enables the detection of SARS-Cov2-specific T cells without preliminary stimulation. T cells labeled by the SARS-Cov2-laden MHC tetramers can be further described by surface markers evaluation or functional tests, such as intracellular cytokine detection [108,109]. Tetramer-positive T cells can also be purified and used for the generation of SARS-Cov2-specific T cell clones [110].

### 6.2. ELISA and ELISPOT

At the beginning of the COVID-19 pandemic, great attention was paid to the diagnosis of humoral immunity. One of the options for the diagnosis of humoral immunity is an enzyme-linked immunosorbent assay (ELISA), a laboratory immunological method for the qualitative or quantitative determination of various compounds based on a specific antigen–antibody reaction [111]. As part of COVID-19 diagnosis, ELISA is mainly used to determine the level of antibodies (IgG, IgM, IgA). Later, several studies have shown that SARS-CoV-2-specific T cells may be a more sensitive marker of a previous COVID-19 infection [58]. In this regard, ELISPOT (Enzyme-Linked ImmunoSpot), a laboratory method used for studying the immune response in humans and animals, has been widely used [112]. Numerous studies have identified that most immunogenic peptides containing a segment of the SARS-CoV-2 T-cell epitope: adhesion peptide (S), nucleopeptide (N), membrane protein, and open reading frame proteins (ORF-3a, ORF-7a) [14]. These recombinant peptides are used to stimulate peripheral blood mononuclear cells to identify effector T cells. This method is widely used to assess the immunological activity of developed vaccines [78,91,100,101,102].

An additional assessment tool is FluoroSpot, which combines ELISpot sensitivity with the ability to measure the secretion of multiple analytes simultaneously, allowing studies of cell populations with different functional profiles [113].

### 6.3. TCR Repertoires Analysis

VDJ recombination of T cell receptor (TCR) coding genes generates highly diverse complementarity determining regions (CDRs), which form a highly diverse functional TCR repertoire. The third CDR (CDR3) of both alpha and beta TCR chains contribute to direct peptide recognition. High-throughput sequencing of CDR3s libraries on T cells from patients during and after SARS-CoV-2 infection identifies repertoire signatures associated with COVID-19, such as repertoire composition, clonal structure, diversity, preferential gene, etc. [114]. Longitudinal repertoire analysis helps to track dynamic changes in the SARS-CoV-2-specific T cell response and investigate the features of pre-existing T-cell memory and formation of new memory clones [115]. TCR repertoire analysis provides new markers for the prediction of disease dynamics and disease outcome [116,117]

TCR repertoire analysis combined with MHC-tetramer staining allows identification of SARS-CoV-2-specific T cell variants, which will provide a basis for the development of effective drugs or vaccines against SARS-CoV-2.

Single-cell TCR-seq combined with differential gene expression analysis helps to obtain an understanding of the main mechanisms of antiviral T cell function during COVID19 infection [118].

### 6.4. CoviDTH and Other Methods

CoviDTH is a delayed response method used for assessing T cell response to S-protein in COVID-19. Currently, studies are being actively performed on the applicability of the method in routine clinical practice and the feasibility of assessing the response to infection to determine a therapeutic strategy. There are positive results regarding these two issues [119,120].

Methods based on measuring the concentration of interferon-γ are also used [92].

In addition, to assess the ability of the immune system to provide an adequate response, a method for determining T and B cell response defects based on the concentration of T cell receptor excision circles (TRECs) and Kappa-deleting recombination excision circles (KRECs) is used. Researchers suggest that TREC circular DNA regions may become new biomarkers of the severity and outcome of COVID-19 [121].

## 7. Discussion

Currently, thanks to the developed scientific infrastructure and the level of modern diagnostics, and taking into account the accumulated experience, the processes of the immune response in SARS-CoV-2 infection are well studied. The normal immune response in this pathology includes both humoral and cellular components.

It is important to note that despite clinicians’ initial focus on antibody formation in determining the management of COVID-19 patients, there is now a growing emphasis on specific cellular immunity against the virus. This is also important because the individual components of cellular immunity are regulated independently: the level of antibodies and cellular immunity often do not correlate with each other.

A significant amount of data accumulated demonstrate that the T cell-mediated immune response to SARS-CoV-2 infection plays a key role in providing anti-viral defense and patients’ recovery. The immune system generates SARS-CoV-2-reactive T cells at the beginning of the infection and control of their dynamic changes provides detailed information about the acute T cell response amplitude and T cell memory formation. This is even more important because it is associated with the prognosis of the patient. Therefore, evaluation of the intensity and duration of the SARS-CoV-2-activated T cell response may be reasonable when appropriate treatment is chosen.

Analysis of the preexisting cross-reactive T-cells, which seem to be linked to mildness of disease, can help to make a rational decision about quarantine measures and vaccination programs regarding each individual patient and at the level of populations.

Findings suggesting that the anti-SARS-CoV-2 T cell response could be crucial for the formation of immune memory against COVID-19 infection combined with the modern methods of antigen-specific T cell clone identification give us a tool for designing T cell-priming vaccines, which would be effective and broadly protective against a range of viral variants.

In this regard, it should be concluded that it is necessary to introduce into practice the assessment of T cell immunity to expand the ability to control infection at the individual and population levels.

## Data Availability

Not applicable.

## References

[1] Zhou P., Yang X.-L., Wang X.-G., Hu B., Zhang L., Zhang W., Si H.-R., Zhu Y., Li B., Huang C.-L. (2020). Addendum: A Pneumonia Outbreak Associated with a New Coronavirus of Probable Bat Origin. Nature.

[2] Lu R., Zhao X., Li J., Niu P., Yang B., Wu H., Wang W., Song H., Huang B., Zhu N. (2020). Genomic Characterisation and Epidemiology of 2019 Novel Coronavirus: Implications for Virus Origins and Receptor Binding. The Lancet.

[3] Quesada J.A., López-Pineda A., Gil-Guillén V.F., Arriero-Marín J.M., Gutiérrez F., Carratala-Munuera C. (2021). Incubation period of COVID-19: A systematic review and meta-analysis. Rev. Clin. Esp..

[4] Sette A., Crotty S. (2021). Adaptive Immunity to SARS-CoV-2 and COVID-19. Cell.

[5] Jordan S.C. (2021). Innate and Adaptive Immune Responses to SARS-CoV-2 in Humans: Relevance to Acquired Immunity and Vaccine Responses. Clin. Exp. Immunol..

[6] Yuki K., Fujiogi M., Koutsogiannaki S. (2020). COVID-19 Pathophysiology: A Review. Clin. Immunol. Orlando Fla.

[7] Cox R.J., Brokstad K.A. (2020). Not Just Antibodies: B Cells and T Cells Mediate Immunity to COVID-19. Nat. Rev. Immunol..

[8] Dan J.M., Mateus J., Kato Y., Hastie K.M., Yu E.D., Faliti C.E., Grifoni A., Ramirez S.I., Haupt S., Frazier A. (2021). Immunological Memory to SARS-CoV-2 Assessed for up to 8 Months after Infection. Science.

[9] Zuo J., Dowell A.C., Pearce H., Verma K., Long H.M., Begum J., Aiano F., Amin-Chowdhury Z., Hoschler K., Brooks T. (2021). Robust SARS-CoV-2-Specific T Cell Immunity Is Maintained at 6 Months Following Primary Infection. Nat. Immunol..

[10] Turvey S.E., Broide D.H. (2010). Chapter 2: Innate Immunity. J. Allergy Clin. Immunol..

[11] Bonilla F.A., Oettgen H.C. (2010). Adaptive Immunity. J. Allergy Clin. Immunol..

[12] Kanellopoulos J.M., Ojcius D.M. (2019). Development of Humoral Immunity. Biomed. J..

[13] Kumar B.V., Connors T.J., Farber D.L. (2018). Human T Cell Development, Localization, and Function throughout Life. Immunity.

[14] Zhao J., Alshukairi A.N., Baharoon S.A., Ahmed W.A., Bokhari A.A., Nehdi A.M., Layqah L.A., Alghamdi M.G., Al Gethamy M.M., Dada A.M. (2017). Recovery from the Middle East Respiratory Syndrome Is Associated with Antibody and T-Cell Responses. Sci. Immunol..

[15] Gu J., Gong E., Zhang B., Zheng J., Gao Z., Zhong Y., Zou W., Zhan J., Wang S., Xie Z. (2005). Multiple Organ Infection and the Pathogenesis of SARS. J. Exp. Med..

[16] Channappanavar R., Fett C., Zhao J., Meyerholz D.K., Perlman S. (2014). Virus-Specific Memory CD8 T Cells Provide Substantial Protection from Lethal Severe Acute Respiratory Syndrome Coronavirus Infection. J. Virol..

[17] Chen J., Lau Y.F., Lamirande E.W., Paddock C.D., Bartlett J.H., Zaki S.R., Subbarao K. (2010). Cellular Immune Responses to Severe Acute Respiratory Syndrome Coronavirus (SARS-CoV) Infection in Senescent BALB/c Mice: CD4+ T Cells Are Important in Control of SARS-CoV Infection. J. Virol..

[18] Zhao J., Zhao J., Perlman S. (2010). T Cell Responses Are Required for Protection from Clinical Disease and for Virus Clearance in Severe Acute Respiratory Syndrome Coronavirus-Infected Mice. J. Virol..

[19] Fan Y.-Y., Huang Z.-T., Li L., Wu M.-H., Yu T., Koup R.A., Bailer R.T., Wu C.-Y. (2009). Characterization of SARS-CoV-Specific Memory T Cells from Recovered Individuals 4 Years after Infection. Arch. Virol..

[20] Chen H., Hou J., Jiang X., Ma S., Meng M., Wang B., Zhang M., Zhang M., Tang X., Zhang F. (2005). Response of Memory CD8+ T Cells to Severe Acute Respiratory Syndrome (SARS) Coronavirus in Recovered SARS Patients and Healthy Individuals. J. Immunol. Baltim. Md 1950.

[21] Yang L.-T., Peng H., Zhu Z.-L., Li G., Huang Z.-T., Zhao Z.-X., Koup R.A., Bailer R.T., Wu C.-Y. (2006). Long-Lived Effector/Central Memory T-Cell Responses to Severe Acute Respiratory Syndrome Coronavirus (SARS-CoV) S Antigen in Recovered SARS Patients. Clin. Immunol. Orlando Fla.

[22] Peng H., Yang L., Wang L., Li J., Huang J., Lu Z., Koup R.A., Bailer R.T., Wu C. (2006). Long-Lived Memory T Lymphocyte Responses against SARS Coronavirus Nucleocapsid Protein in SARS-Recovered Patients. Virology.

[23] Yang L., Peng H., Zhu Z., Li G., Huang Z., Zhao Z., Koup R.A., Bailer R.T., Wu C. (2007). Persistent Memory CD4+ and CD8+ T-Cell Responses in Recovered Severe Acute Respiratory Syndrome (SARS) Patients to SARS Coronavirus M Antigen. J. Gen. Virol..

[24] Guan W.D., Mok C.K.P., Chen Z.L., Feng L.Q., Li Z.T., Huang J.C., Ke C.W., Deng X., Ling Y., Wu S.G. (2015). Characteristics of Traveler with Middle East Respiratory Syndrome, China, 2015. Emerg. Infect. Dis..

[25] Ng O.-W., Chia A., Tan A.T., Jadi R.S., Leong H.N., Bertoletti A., Tan Y.-J. (2016). Memory T Cell Responses Targeting the SARS Coronavirus Persist up to 11 Years Post-Infection. Vaccine.

[26] Oh H.-L.J., Chia A., Chang C.X.L., Leong H.N., Ling K.L., Grotenbreg G.M., Gehring A.J., Tan Y.J., Bertoletti A. (2011). Engineering T Cells Specific for a Dominant Severe Acute Respiratory Syndrome Coronavirus CD8 T Cell Epitope. J. Virol..

[27] Tang F., Quan Y., Xin Z.-T., Wrammert J., Ma M.-J., Lv H., Wang T.-B., Yang H., Richardus J.H., Liu W. (2011). Lack of Peripheral Memory B Cell Responses in Recovered Patients with Severe Acute Respiratory Syndrome: A Six-Year Follow-up Study. J. Immunol. Baltim. Md 1950.

[28] Min C.-K., Cheon S., Ha N.-Y., Sohn K.M., Kim Y., Aigerim A., Shin H.M., Choi J.-Y., Inn K.-S., Kim J.-H. (2016). Comparative and Kinetic Analysis of Viral Shedding and Immunological Responses in MERS Patients Representing a Broad Spectrum of Disease Severity. Sci. Rep..

[29] Liu W.J., Zhao M., Liu K., Xu K., Wong G., Tan W., Gao G.F. (2017). T-Cell Immunity of SARS-CoV: Implications for Vaccine Development against MERS-CoV. Antiviral Res..

[30] Wang W.L., Wang H.J., Deng Y., Song T., Lan J.M., Wu G.Z., Ke C.W., Tan W.J. (2016). Serological Study of An Imported Case of Middle East Respiratory Syndrome and His Close Contacts in China, 2015. Biomed. Environ. Sci. BES.

[31] Kapoor M., Pringle K., Kumar A., Dearth S., Liu L., Lovchik J., Perez O., Pontones P., Richards S., Yeadon-Fagbohun J. (2014). Clinical and Laboratory Findings of the First Imported Case of Middle East Respiratory Syndrome Coronavirus to the United States. Clin. Infect. Dis. Off. Publ. Infect. Dis. Soc. Am..

[32] Corman V.M., Albarrak A.M., Omrani A.S., Albarrak M.M., Farah M.E., Almasri M., Muth D., Sieberg A., Meyer B., Assiri A.M. (2016). Viral Shedding and Antibody Response in 37 Patients With Middle East Respiratory Syndrome Coronavirus Infection. Clin. Infect. Dis. Off. Publ. Infect. Dis. Soc. Am..

[33] Park W.B., Perera R.A.P.M., Choe P.G., Lau E.H.Y., Choi S.J., Chun J.Y., Oh H.S., Song K.-H., Bang J.H., Kim E.S. (2015). Kinetics of Serologic Responses to MERS Coronavirus Infection in Humans, South Korea. Emerg. Infect. Dis..

[34] Faure E., Poissy J., Goffard A., Fournier C., Kipnis E., Titecat M., Bortolotti P., Martinez L., Dubucquoi S., Dessein R. (2014). Distinct Immune Response in Two MERS-CoV-Infected Patients: Can We Go from Bench to Bedside?. PloS ONE.

[35] Al-Abdallat M.M., Payne D.C., Alqasrawi S., Rha B., Tohme R.A., Abedi G.R., Al Nsour M., Iblan I., Jarour N., Farag N.H. (2014). Hospital-Associated Outbreak of Middle East Respiratory Syndrome Coronavirus: A Serologic, Epidemiologic, and Clinical Description. Clin. Infect. Dis. Off. Publ. Infect. Dis. Soc. Am..

[36] Alshukairi A.N., Khalid I., Ahmed W.A., Dada A.M., Bayumi D.T., Malic L.S., Althawadi S., Ignacio K., Alsalmi H.S., Al-Abdely H.M. (2016). Antibody Response and Disease Severity in Healthcare Worker MERS Survivors. Emerg. Infect. Dis..

[37] Arabi Y.M., Hajeer A.H., Luke T., Raviprakash K., Balkhy H., Johani S., Al-Dawood A., Al-Qahtani S., Al-Omari A., Al-Hameed F. (2016). Feasibility of Using Convalescent Plasma Immunotherapy for MERS-CoV Infection, Saudi Arabia. Emerg. Infect. Dis..

[38] Wang S.-F., Chen K.-H., Chen M., Li W.-Y., Chen Y.-J., Tsao C.-H., Yen M., Huang J.C., Chen Y.-M.A. (2011). Human-Leukocyte Antigen Class I Cw 1502 and Class II DR 0301 Genotypes Are Associated with Resistance to Severe Acute Respiratory Syndrome (SARS) Infection. Viral Immunol..

[39] Giamarellos-Bourboulis E.J., Netea M.G., Rovina N., Akinosoglou K., Antoniadou A., Antonakos N., Damoraki G., Gkavogianni T., Adami M.-E., Katsaounou P. (2020). Complex Immune Dysregulation in COVID-19 Patients with Severe Respiratory Failure. Cell Host Microbe.

[40] Li C.K., Wu H., Yan H., Ma S., Wang L., Zhang M., Tang X., Temperton N.J., Weiss R.A., Brenchley J.M. (2008). T Cell Responses to Whole SARS Coronavirus in Humans. J. Immunol. Baltim. Md 1950.

[41] Lipsitch M., Grad Y.H., Sette A., Crotty S. (2020). Cross-Reactive Memory T Cells and Herd Immunity to SARS-CoV-2. Nat. Rev. Immunol..

[42] Sette A., Crotty S. (2020). Pre-Existing Immunity to SARS-CoV-2: The Knowns and Unknowns. Nat. Rev. Immunol..

[43] Anderson E.M., Goodwin E.C., Verma A., Arevalo C.P., Bolton M.J., Weirick M.E., Gouma S., McAllister C.M., Christensen S.R., Weaver J. (2021). Seasonal Human Coronavirus Antibodies Are Boosted upon SARS-CoV-2 Infection but Not Associated with Protection. Cell.

[44] Yamaguchi T., Shinagawa T., Kobata H., Nakagawa H. (2021). Immunity against Seasonal Human Coronavirus OC43 Mitigates Fatal Deterioration of COVID-19. Int. J. Infect. Dis..

[45] Shah V.K., Firmal P., Alam A., Ganguly D., Chattopadhyay S. (2020). Overview of Immune Response During SARS-CoV-2 Infection: Lessons From the Past. Front. Immunol..

[46] Blanco-Melo D., Nilsson-Payant B.E., Liu W.-C., Uhl S., Hoagland D., Møller R., Jordan T.X., Oishi K., Panis M., Sachs D. (2020). Imbalanced Host Response to SARS-CoV-2 Drives Development of COVID-19. Cell.

[47] Zheng H.-Y., Zhang M., Yang C.-X., Zhang N., Wang X.-C., Yang X.-P., Dong X.-Q., Zheng Y.-T. (2020). Elevated Exhaustion Levels and Reduced Functional Diversity of T Cells in Peripheral Blood May Predict Severe Progression in COVID-19 Patients. Cell. Mol. Immunol..

[48] Qin C., Zhou L., Hu Z., Zhang S., Yang S., Tao Y., Xie C., Ma K., Shang K., Wang W. (2020). Dysregulation of Immune Response in Patients With Coronavirus 2019 (COVID-19) in Wuhan, China. Clin. Infect. Dis. Off. Publ. Infect. Dis. Soc. Am..

[49] Li G., Chen X., Xu A. (2003). Profile of Specific Antibodies to the SARS-Associated Coronavirus. N. Engl. J. Med..

[50] Gudbjartsson D.F., Norddahl G.L., Melsted P., Gunnarsdottir K., Holm H., Eythorsson E., Arnthorsson A.O., Helgason D., Bjarnadottir K., Ingvarsson R.F. (2020). Humoral Immune Response to SARS-CoV-2 in Iceland. N. Engl. J. Med..

[51] Wajnberg A., Amanat F., Firpo A., Altman D.R., Bailey M.J., Mansour M., McMahon M., Meade P., Mendu D.R., Muellers K. (2020). Robust Neutralizing Antibodies to SARS-CoV-2 Infection Persist for Months. Science.

[52] Molodtsov I.A., Kegeles E., Mitin A.N., Mityaeva O., Musatova O.E., Panova A.E., Pashenkov M.V., Peshkova I.O., Almaqdad A., Asaad W. (2021). A Prospective Study of the Protective Effect of SARS-CoV-2–Specific Antibodies and T Cells in Moscow Residents. MedRxiv.

[53] Tan W., Lu Y., Zhang J., Wang J., Dan Y., Tan Z., He X., Qian C., Sun Q., Hu Q. (2020). Viral Kinetics and Antibody Responses in Patients with COVID-19. Medrxiv.

[54] Zhang Y., Xu J., Jia R., Yi C., Gu W., Liu P., Dong X., Zhou H., Shang B., Cheng S. (2020). Protective Humoral Immunity in SARS-CoV-2 Infected Pediatric Patients. Cell. Mol. Immunol..

[55] Liu J., Wu P., Gao F., Qi J., Kawana-Tachikawa A., Xie J., Vavricka C.J., Iwamoto A., Li T., Gao G.F. (2010). Novel Immunodominant Peptide Presentation Strategy: A Featured HLA-A*2402-Restricted Cytotoxic T-Lymphocyte Epitope Stabilized by Intrachain Hydrogen Bonds from Severe Acute Respiratory Syndrome Coronavirus Nucleocapsid Protein. J. Virol..

[56] Grifoni A., Weiskopf D., Ramirez S.I., Mateus J., Dan J.M., Moderbacher C.R., Rawlings S.A., Sutherland A., Premkumar L., Jadi R.S. (2020). Targets of T Cell Responses to SARS-CoV-2 Coronavirus in Humans with COVID-19 Disease and Unexposed Individuals. Cell.

[57] Rydyznski Moderbacher C., Ramirez S.I., Dan J.M., Grifoni A., Hastie K.M., Weiskopf D., Belanger S., Abbott R.K., Kim C., Choi J. (2020). Antigen-Specific Adaptive Immunity to SARS-CoV-2 in Acute COVID-19 and Associations with Age and Disease Severity. Cell.

[58] Sekine T., Perez-Potti A., Rivera-Ballesteros O., Strålin K., Gorin J.-B., Olsson A., Llewellyn-Lacey S., Kamal H., Bogdanovic G., Muschiol S. (2020). Robust T Cell Immunity in Convalescent Individuals with Asymptomatic or Mild COVID-19. Cell.

[59] Chen Z., John Wherry E. (2020). T Cell Responses in Patients with COVID-19. Nat. Rev. Immunol..

[60] Tan A.T., Linster M., Tan C.W., Le Bert N., Chia W.N., Kunasegaran K., Zhuang Y., Tham C.Y.L., Chia A., Smith G.J.D. (2021). Early Induction of Functional SARS-CoV-2-Specific T Cells Associates with Rapid Viral Clearance and Mild Disease in COVID-19 Patients. Cell Rep..

[61] Gutmann C., Takov K., Burnap S.A., Singh B., Ali H., Theofilatos K., Reed E., Hasman M., Nabeebaccus A., Fish M. (2021). SARS-CoV-2 RNAemia and Proteomic Trajectories Inform Prognostication in COVID-19 Patients Admitted to Intensive Care. Nat. Commun..

[62] Marcotte H., Piralla A., Zuo F., Du L., Cassaniti I., Wan H., Kumagai-Braesh M., Andréll J., Percivalle E., Sammartino J.C. (2022). Immunity to SARS-CoV-2 up to 15 Months after Infection. Iscience.

[63] Cameron M.J., Bermejo-Martin J.F., Danesh A., Muller M.P., Kelvin D.J. (2008). Human Immunopathogenesis of Severe Acute Respiratory Syndrome (SARS). Virus Res..

[64] Chien J.-Y., Hsueh P.-R., Cheng W.-C., Yu C.-J., Yang P.-C. (2006). Temporal Changes in Cytokine/Chemokine Profiles and Pulmonary Involvement in Severe Acute Respiratory Syndrome. Respirol. Carlton Vic.

[65] Shrotri M., van Schalkwyk M.C.I., Post N., Eddy D., Huntley C., Leeman D., Rigby S., Williams S.V., Bermingham W.H., Kellam P. (2021). T Cell Response to SARS-CoV-2 Infection in Humans: A Systematic Review. PLoS ONE.

[66] Yu K.K.Q., Fischinger S., Smith M.T., Atyeo C., Cizmeci D., Wolf C.R., Layton E.D., Logue J.K., Aguilar M.S., Shuey K. (2020). T Cell and Antibody Functional Correlates of Severe COVID-19. MedRxiv Prepr. Serv. Health Sci..

[67] Wang Z., Yang X., Zhou Y., Sun J., Liu X., Zhang J., Mei X., Zhong J., Zhao J., Ran P. (2020). COVID-19 Severity Correlates with Weaker T-Cell Immunity, Hypercytokinemia, and Lung Epithelium Injury. Am. J. Respir. Crit. Care Med..

[68] Takahashi T., Ellingson M.K., Wong P., Israelow B., Lucas C., Klein J., Silva J., Mao T., Oh J.E., Tokuyama M. (2020). Sex Differences in Immune Responses That Underlie COVID-19 Disease Outcomes. Nature.

[69] Ciarambino T., Para O., Giordano M. (2021). Immune System and COVID-19 by Sex Differences and Age. Womens Health.

[70] Cohen C.A., Li A.P.Y., Hachim A., Hui D.S.C., Kwan M.Y.W., Tsang O.T.Y., Chiu S.S., Chan W.H., Yau Y.S., Kavian N. (2021). SARS-CoV-2 Specific T Cell Responses Are Lower in Children and Increase with Age and Time after Infection. Nat. Commun..

[71] Bajaj V., Gadi N., Spihlman A.P., Wu S.C., Choi C.H., Moulton V.R. (2021). Aging, Immunity, and COVID-19: How Age Influences the Host Immune Response to Coronavirus Infections?. Front. Physiol..

[72] Pain C.E., Felsenstein S., Cleary G., Mayell S., Conrad K., Harave S., Duong P., Sinha I., Porter D., Hedrich C.M. (2020). Novel Paediatric Presentation of COVID-19 with ARDS and Cytokine Storm Syndrome without Respiratory Symptoms. Lancet Rheumatol..

[73] (2021). Very-Low-Carbohydrate Diet Enhances Human T-Cell Immunity through Immunometabolic Reprogramming. EMBO Mol. Med..

[74] Calder P.C. (2021). Nutrition and Immunity: Lessons for COVID-19. Eur. J. Clin. Nutr..

[75] Calder P.C. (2020). Nutrition, Immunity and COVID-19. BMJ Nutr. Prev. Health.

[76] Zelzer S., Prüller F., Curcic P., Sloup Z., Holter M., Herrmann M., Mangge H. (2021). Vitamin D Metabolites and Clinical Outcome in Hospitalized COVID-19 Patients. Nutrients.

[77] Mangge H., Herrmann M., Meinitzer A., Pailer S., Curcic P., Sloup Z., Holter M., Prüller F. (2021). Increased Kynurenine Indicates a Fatal Course of COVID-19. Antioxidants.

[78] Polack F.P., Thomas S.J., Kitchin N., Absalon J., Gurtman A., Lockhart S., Perez J.L., Pérez Marc G., Moreira E.D., Zerbini C. (2020). Safety and Efficacy of the BNT162b2 MRNA Covid-19 Vaccine. N. Engl. J. Med..

[79] Logunov D.Y., Dolzhikova I.V., Shcheblyakov D.V., Tukhvatulin A.I., Zubkova O.V., Dzharullaeva A.S., Kovyrshina A.V., Lubenets N.L., Grousova D.M., Erokhova A.S. (2021). Safety and Efficacy of an RAd26 and RAd5 Vector-Based Heterologous Prime-Boost COVID-19 Vaccine: An Interim Analysis of a Randomised Controlled Phase 3 Trial in Russia. Lancet Lond. Engl..

[80] Shinde V., Bhikha S., Hoosain Z., Archary M., Bhorat Q., Fairlie L., Lalloo U., Masilela M.S.L., Moodley D., Hanley S. (2021). Efficacy of the NVX-CoV2373 Covid-19 Vaccine Against the B.1.351 Variant. N. Engl. J. Med..

[81] Thompson M.G., Burgess J.L., Naleway A.L., Tyner H., Yoon S.K., Meece J., Olsho L.E.W., Caban-Martinez A.J., Fowlkes A.L., Lutrick K. (2021). Prevention and Attenuation of Covid-19 with the BNT162b2 and MRNA-1273 Vaccines. N. Engl. J. Med..

[82] Le Bert N., Tan A.T., Kunasegaran K., Tham C.Y., Hafezi M., Chia A., Chng M.H.Y., Lin M., Tan N., Linster M. (2020). SARS-CoV-2-Specific T Cell Immunity in Cases of COVID-19 and SARS, and Uninfected Controls. Nature.

[83] Madhi S.A., Baillie V., Cutland C.L., Voysey M., Koen A.L., Fairlie L., Padayachee S.D., Dheda K., Barnabas S.L., Bhorat Q.E. (2021). Efficacy of the ChAdOx1 NCoV-19 Covid-19 Vaccine against the B.1.351 Variant. N. Engl. J. Med..

[84] Altmann D.M., Boyton R.J., Beale R. (2021). Immunity to SARS-CoV-2 Variants of Concern. Science.

[85] Reynolds C.J., Pade C., Gibbons J.M., Butler D.K., Otter A.D., Menacho K., Fontana M., Smit A., Sackville-West J.E., Cutino-Moguel T. (2021). Prior SARS-CoV-2 Infection Rescues B and T Cell Responses to Variants after First Vaccine Dose. Science.

[86] Woldemeskel B.A., Garliss C.C., Blankson J.N. (2021). SARS-CoV-2 MRNA Vaccines Induce Broad CD4+ T Cell Responses That Recognize SARS-CoV-2 Variants and HCoV-NL63. J. Clin. Investig..

[87] Geers D., Shamier M.C., Bogers S., den Hartog G., Gommers L., Nieuwkoop N.N., Schmitz K.S., Rijsbergen L.C., van Osch J.A.T., Dijkhuizen E. (2021). SARS-CoV-2 Variants of Concern Partially Escape Humoral but Not T-Cell Responses in COVID-19 Convalescent Donors and Vaccinees. Sci. Immunol..

[88] Alter G., Yu J., Liu J., Chandrashekar A., Borducchi E.N., Tostanoski L.H., McMahan K., Jacob-Dolan C., Martinez D.R., Chang A. (2021). Immunogenicity of Ad26.COV2.S Vaccine against SARS-CoV-2 Variants in Humans. Nature.

[89] Stanevich O., Alekseeva E., Sergeeva M., Fadeev A., Komissarova K., Ivanova A., Simakova T., Vasilyev K., Shurygina A.-P., Stukova M. (2021). SARS-CoV-2 Escape from Cytotoxic T Cells during Long-Term COVID-19. Nature Portfolio.

[90] Painter M.M., Mathew D., Goel R.R., Apostolidis S.A., Pattekar A., Kuthuru O., Baxter A.E., Herati R.S., Oldridge D.A., Gouma S. (2021). Rapid Induction of Antigen-Specific CD4+ T Cells Is Associated with Coordinated Humoral and Cellular Immunity to SARS-CoV-2 MRNA Vaccination. Immunity.

[91] Sahin U., Muik A., Vogler I., Derhovanessian E., Kranz L.M., Vormehr M., Quandt J., Bidmon N., Ulges A., Baum A. (2021). BNT162b2 Vaccine Induces Neutralizing Antibodies and Poly-Specific T Cells in Humans. Nature.

[92] Tan A.T., Lim J.M.E., Bert N.L., Kunasegaran K., Chia A., Qui M.D.C., Tan N., Chia W.N., de Alwis R., Ying D. (2021). Rapid Measurement of SARS-CoV-2 Spike T Cells in Whole Blood from Vaccinated and Naturally Infected Individuals. J. Clin. Investig..

[93] Mateus J., Dan J.M., Zhang Z., Rydyznski Moderbacher C., Lammers M., Goodwin B., Sette A., Crotty S., Weiskopf D. (2021). Low-Dose MRNA-1273 COVID-19 Vaccine Generates Durable Memory Enhanced by Cross-Reactive T Cells. Science.

[94] Goel R.R., Painter M.M., Apostolidis S.A., Mathew D., Meng W., Rosenfeld A.M., Lundgreen K.A., Reynaldi A., Khoury D.S., Pattekar A. (2021). MRNA Vaccines Induce Durable Immune Memory to SARS-CoV-2 and Variants of Concern. Science.

[95] Wilson E.A., Hirneise G., Singharoy A., Anderson K.S. (2020). Total Predicted MHC-I Epitope Load Is Inversely Associated with Population Mortality from SARS-COV-2. Cell Rep. Med..

[96] Prendecki M., Clarke C., Brown J., Cox A., Gleeson S., Guckian M., Randell P., Pria A.D., Lightstone L., Xu X.-N. (2021). Effect of Previous SARS-CoV-2 Infection on Humoral and T-Cell Responses to Single-Dose BNT162b2 Vaccine. Lancet Lond. Engl..

[97] Collier D.A., Ferreira I.A.T.M., Kotagiri P., Datir R.P., Lim E.Y., Touizer E., Meng B., Abdullahi A., Elmer A., Kingston N. (2021). Age-Related Immune Response Heterogeneity to SARS-CoV-2 Vaccine BNT162b2. Nature.

[98] Apostolidis S.A., Kakara M., Painter M.M., Goel R.R., Mathew D., Lenzi K., Rezk A., Patterson K.R., Espinoza D.A., Kadri J.C. (2021). Altered Cellular and Humoral Immune Responses Following SARS-CoV-2 MRNA Vaccination in Patients with Multiple Sclerosis on Anti-CD20 Therapy. Nat. Med..

[99] Reynolds C.J., Swadling L., Gibbons J.M., Pade C., Jensen M.P., Diniz M.O., Schmidt N.M., Butler D.K., Amin O.E., Bailey S.N.L. (2020). Discordant Neutralizing Antibody and T Cell Responses in Asymptomatic and Mild SARS-CoV-2 Infection. Sci. Immunol..

[100] Deng Y., Li Y., Yang R., Tan W. (2021). SARS-CoV-2-Specific T Cell Immunity to Structural Proteins in Inactivated COVID-19 Vaccine Recipients. Cell. Mol. Immunol..

[101] Zhu F.-C., Li Y.-H., Guan X.-H., Hou L.-H., Wang W.-J., Li J.-X., Wu S.-P., Wang B.-S., Wang Z., Wang L. (2020). Safety, Tolerability, and Immunogenicity of a Recombinant Adenovirus Type-5 Vectored COVID-19 Vaccine: A Dose-Escalation, Open-Label, Non-Randomised, First-in-Human Trial. Lancet.

[102] Folegatti P.M., Ewer K.J., Aley P.K., Angus B., Becker S., Belij-Rammerstorfer S., Bellamy D., Bibi S., Bittaye M., Clutterbuck E.A. (2020). Safety and Immunogenicity of the ChAdOx1 NCoV-19 Vaccine against SARS-CoV-2: A Preliminary Report of a Phase 1/2, Single-Blind, Randomised Controlled Trial. Lancet.

[103] Borobia A.M., Carcas A.J., Pérez-Olmeda M., Castaño L., Bertran M.J., García-Pérez J., Campins M., Portolés A., González-Pérez M., Morales M.T.G. (2021). Immunogenicity and Reactogenicity of BNT162b2 Booster in ChAdOx1-S-Primed Participants (CombiVacS): A Multicentre, Open-Label, Randomised, Controlled, Phase 2 Trial. Lancet.

[104] Schmidt T., Klemis V., Schub D., Mihm J., Hielscher F., Marx S., Abu-Omar A., Ziegler L., Guckelmus C., Urschel R. (2021). Immunogenicity and Reactogenicity of Heterologous ChAdOx1 NCoV-19/MRNA Vaccination. Nat. Med..

[105] Oberhardt V., Luxenburger H., Kemming J., Schulien I., Ciminski K., Giese S., Csernalabics B., Lang-Meli J., Janowska I., Staniek J. (2021). Rapid and Stable Mobilization of CD8+ T Cells by SARS-CoV-2 MRNA Vaccine. Nature.

[106] Dennehy K.M., Löll E., Dhillon C., Classen J.-M., Warm T.D., Schuierer L., Hyhlik-Dürr A., Römmele C., Gosslau Y., Kling E. (2021). Comparison of the Development of SARS-Coronavirus-2-Specific Cellular Immunity, and Central Memory CD4+ T-Cell Responses Following Infection versus Vaccination. Vaccines.

[107] Soni N., Pai P., Krishna Kumar G.R., Prasad V., Dasgupta S., Bhadra B. (2020). A Flow Virometry Process Proposed for Detection of SARS-CoV-2 and Large-Scale Screening of COVID-19 Cases. Future Virol..

[108] Chang J. (2021). MHC Multimer: A Molecular Toolbox for Immunologists. Mol. Cells.

[109] Kared H., Redd A.D., Bloch E.M., Bonny T.S., Sumatoh H., Kairi F., Carbajo D., Abel B., Newell E.W., Bettinotti M.P. (2020). CD8+ T Cell Responses in Convalescent COVID-19 Individuals Target Epitopes from the Entire SARS-CoV-2 Proteome and Show Kinetics of Early Differentiation. Immunology.

[110] Bartolo L., Afroz S., Pan Y.-G., Xu R., Williams L., Lin C.-F., Friedman E.S., Gimotty P.A., Wu G.D., Su L.F. (2021). SARS-CoV-2-Specific T Cells in Unexposed Adults Display Broad Trafficking Potential and Cross-React with Commensal Antigens. Immunology.

[111] Егoрoв  A.M. (1991). Теoрия и практика иммунoферментнoгo анализа.

[112] Wyllie D., Jones H.E., Mulchandani R., Trickey A., Taylor-Phillips S., Brooks T., Charlett A., Ades A.E., Investigators E.-H., Moore P. (2021). SARS-CoV-2 Responsive T Cell Numbers and Anti-Spike IgG Levels Are Both Associated with Protection from COVID-19: A Prospective Cohort Study in Keyworkers. medRxiv.

[113] Ahlborg N., Axelsson B. (2012). Dual- and Triple-Color Fluorospot. Methods Mol. Biol. Clifton NJ.

[114] Chang C.-M., Feng P., Wu T.-H., Alachkar H., Lee K.-Y., Chang W.-C. (2021). Profiling of T Cell Repertoire in SARS-CoV-2-Infected COVID-19 Patients Between Mild Disease and Pneumonia. J. Clin. Immunol..

[115] Minervina A.A., Komech E.A., Titov A., Bensouda Koraichi M., Rosati E., Mamedov I.Z., Franke A., Efimov G.A., Chudakov D.M., Mora T. (2021). Longitudinal High-Throughput TCR Repertoire Profiling Reveals the Dynamics of T-Cell Memory Formation after Mild COVID-19 Infection. eLife.

[116] Park J.J., Lee K.A.V., Lam S.Z., Chen S. (2021). T Cell Receptor Repertoire Signatures Associated with COVID-19 Severity. Bioinformatics.

[117] Wen W., Su W., Tang H., Le W., Zhang X., Zheng Y., Liu X., Xie L., Li J., Ye J. (2020). Immune Cell Profiling of COVID-19 Patients in the Recovery Stageby Single-Cell Sequencing. Cell Discov..

[118] Luo L., Liang W., Pang J., Xu G., Chen Y., Guo X., Wang X., Zhao Y., Lai Y., Liu Y. (2021). Dynamics of TCR Repertoire and T Cell Function in COVID-19 Convalescent Individuals. Cell Discov..

[119] Barrios Y., Franco A., Sanchez-Machin I., Poza-Guedes P., Gonzalez-Perez R., Matheu V. (2021). A Novel Application of Delayed-Type Hipersensitivity Reaction to Measure Cellular Immune Response in SARS-CoV-2 Exposed Individuals. Clin. Immunol. Orlando Fla.

[120] Barrios Y., Franco A., Sánchez-Machín I., Poza-Guedes P., González-Pérez R., Matheu V. (2021). The Beauty of Simplicity: Delayed-Type Hypersensitivity Reaction to Measure Cellular Immune Responses in RNA-SARS-Cov-2 Vaccinated Individuals. Vaccines.

[121] Cuvelier P., Roux H., Couëdel-Courteille A., Dutrieux J., Naudin C., Charmeteau de Muylder B., Cheynier R., Squara P., Marullo S. (2021). Protective Reactive Thymus Hyperplasia in COVID-19 Acute Respiratory Distress Syndrome. Crit. Care.

